# Antiplatelet therapy in cardiovascular disease: Current status and future directions

**DOI:** 10.1111/bcp.15221

**Published:** 2022-02-03

**Authors:** Gabriella Passacquale, Pankaj Sharma, Divaka Perera, Albert Ferro

**Affiliations:** ^1^ School of Cardiovascular Medicine and Sciences, British Heart Foundation Centre of Research Excellence King's College London London UK; ^2^ Institute of Cardiovascular Research, Royal Holloway University of London Egham Surrey UK

**Keywords:** antiplatelet agents, cardiovascular disease, thrombosis

## Abstract

Antiplatelet medications remain a cornerstone of therapy for atherosclerotic cardiovascular and cerebrovascular diseases. In primary prevention (patients with cardiovascular risk factors but no documented events, symptoms or angiographic disease), there is little evidence of benefit of any antiplatelet therapy, and such therapy carries the risk of excess bleeding. Where there is documented disease (secondary prevention), stable patients benefit from long‐term antiplatelet monotherapy, aspirin being first choice in those with coronary heart disease and clopidogrel in those with cerebrovascular disease; moreover, recent evidence shows that low‐dose rivaroxaban in combination with aspirin confers added benefit, in patients with stable cardiovascular and peripheral arterial disease. In patients with acute cerebrovascular disease, aspirin combined with clopidogrel reduces subsequent risk, while in acute coronary syndrome, dual antiplatelet therapy comprising aspirin and a P2Y_12_ inhibitor (clopidogrel, prasugrel or ticagrelor) confers greater protection than aspirin monotherapy, with prasugrel and ticagrelor offering greater antiplatelet efficacy with faster onset of action than clopidogrel. Although greater antiplatelet efficacy is advantageous in preventing thrombotic events, this must be tempered by increased risk of bleeding, which may be a particular issue in certain patient groups, as will be discussed. We will also discuss possible future approaches to personalisation of antiplatelet therapy.

## 1 INTRODUCTION

The cyclooxygenase (COX) inhibitor aspirin was first introduced into cardiovascular prophylaxis in the 1980s, and the subsequent introduction of the adenosine purinergic (ADP) receptor P2Y_12_ inhibitors not only offered an alternative for aspirin‐intolerant patients, but also the potential for high intensity platelet inhibition due to simultaneous blockade of COX and ADP‐dependent pathways. However, the more potent antithrombotic action from blockade of both pathways also carries a higher risk of bleeding complications; and although concomitant proton‐pump inhibitor therapy will help to prevent gastrointestinal haemorrhage in patients with acid peptic disease, it will not abolish the risk, nor will it impact bleeding at other sites. Much research has therefore centred around the appropriate use of dual antiplatelet therapy (DAPT) to establish both optimal drug combination and ideal duration of treatment, aiming for a net positive balance between beneficial (antithrombotic) and detrimental (bleeding) effects.

Here we aim to clarify for clinicians the evidence for the use of aspirin and P2Y_12_ inhibitors in different clinical situations, either as mono or dual therapy. We will also touch on the place of anticoagulation on top of antiplatelet therapy in the context of atherosclerotic diseases. Finally, we will consider whether personalised approaches to antiplatelet therapy may be useful for maximising benefit/risk ratio.

## 2 KEY PHARMACOLOGY

**FIGURE 1:**
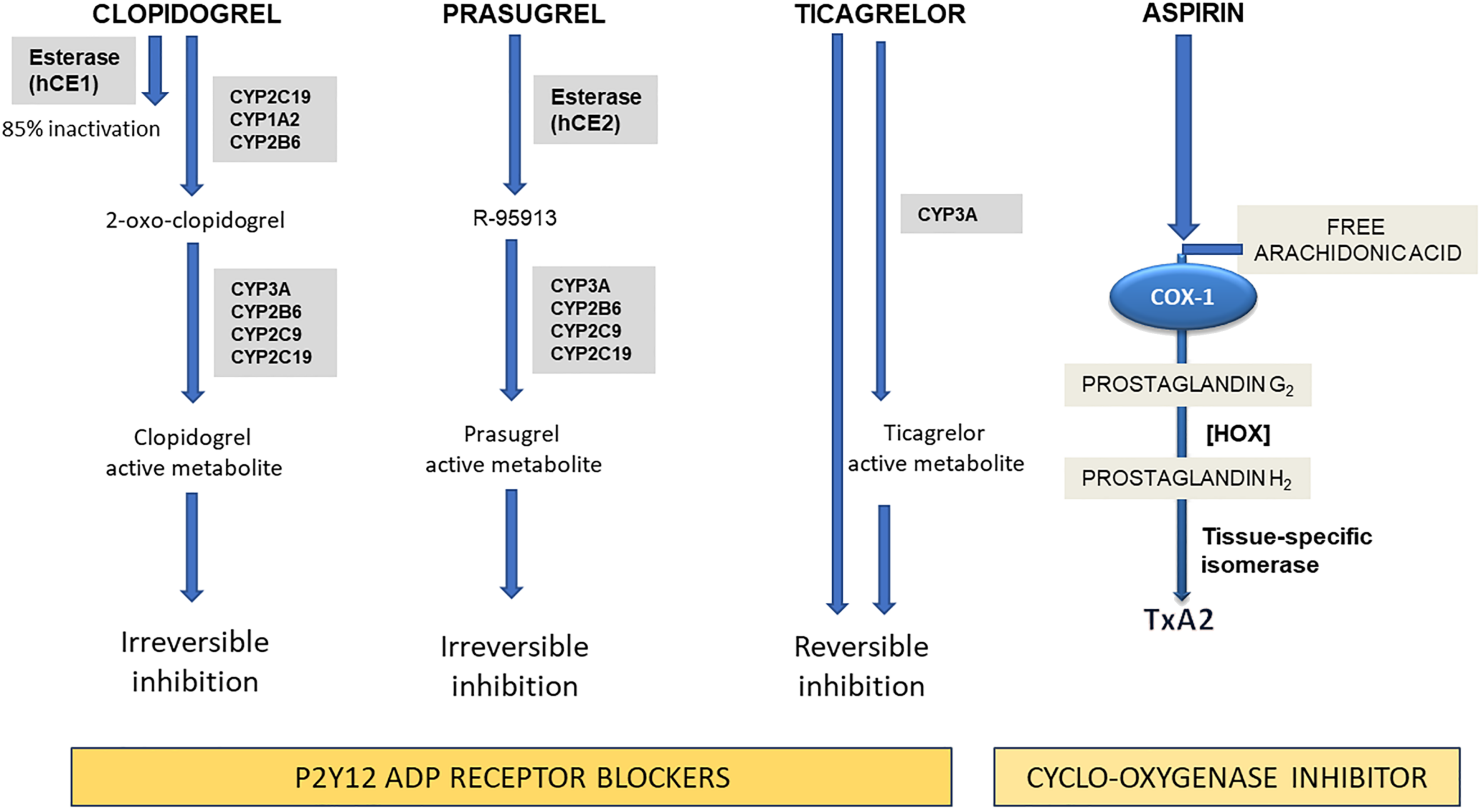
Antiplatelet drug mechanisms of action. The thienopyridines clopidogrel and prasugrel prevent ADP from binding its specific P2Y_12_ receptor and cause its irreversible inhibition; ticagrelor exerts reversible P2Y_12_ receptor antagonism. While clopidogrel and prasugrel require hepatic metabolism to produce the active drug metabolite, ticagrelor is not a prodrug and has a direct inhibitory action, although additionally undergoing a cytochrome‐dependent oxidation that also produces an active metabolite contributing to the pharmacological effect. Aspirin irreversibly blocks the enzymatic activity of cyclooxygenase‐1 (COX‐1), which is a key enzyme in the metabolism of arachidonic acid to produce prostanoids. COX‐1 converts arachidonic acid to the unstable intermediate prostaglandin G2 (PGG2). Further metabolism of PGG2 by hydroperoxidases (HOX) leads to prostaglandin H2 synthesis that is finally converted into prostanoids by tissue‐specific isomerases (platelets mainly contain thromboxane A2 [TxA2] synthase resulting in production and release of TxA2). By acting on COX‐1, aspirin reduces TxA2‐dependent platelet activation. CYP: cytochrome P450. hCE: human carboxylesterase

The principal agents in clinical use are aspirin (acetylsalicylic acid) and the P2Y_12_ receptor inhibitor drugs clopidogrel, prasugrel and ticagrelor (Figure 1).

Aspirin acetylates a critical serine residue in the active site of the COX‐1 isoenzyme, causing irreversible inhibition of platelet COX activity with consequent suppression of thromboxane‐dependent platelet activation.

The P2Y_12_ receptor on the platelet surface binds ADP, which, via the mediation of Gi protein, activates the platelet glycoprotein IIb/IIIa receptor resulting in enhanced platelet degranulation, thromboxane production and platelet aggregation. The first P2Y_12_ inhibitor developed for clinical use, ticlopidine, a thienopyridine derivative, was rapidly replaced by the second‐generation thienopyridine clopidogrel in view of its more favourable safety profile.^1^ Subsequently, the third generation thienopyridine prasugrel was developed, which exhibited the advantages of increased efficacy and more predictable metabolism of prodrug to active drug.^2^ Both clopidogrel and prasugrel require oxidation by hepatic cytochrome P450 to be converted to their active metabolites. The active molecules selectively and irreversibly bind P2Y_12_ receptors on platelets, thus preventing ADP‐dependent platelet activation.^3^

Ticagrelor is a reversible P2Y_12_ inhibitor of the cyclo‐pentyl‐triazolo‐pyrimidine class that, unlike the thienopyridines, is active in its own right and does not require hepatic metabolism to exert its pharmacological action, although cytochrome‐mediated oxidation of the drug is extensive and leads to the formation of an active metabolite that also contributes to the therapeutic effect.^4^ It therefore exhibits faster offset of effect. A common adverse event is dyspnoea which represents the most frequent cause of therapy discontinuation (physician‐recommended drug cessation), interruption (temporary drug cessation) or disruption (unplanned cessation due to adverse events or lack of adherence).^5^

As this review centres on antiplatelet therapy, we will not discuss the pharmacology of the direct oral anticoagulants (DOACs), which has been reviewed in detail recently.^6^ Nevertheless, it is pertinent to mention the DOACs here, because of much recent interest in the concomitant use of DOACs with antiplatelets for cardiovascular prevention, largely thanks to the results of the COMPASS trial (Table 1),^12^ which will be discussed below.

**TABLE 1:** Major randomised controlled clinical trials testing antiplatelet strategies in the secondary prophylaxis of cardiac and peripheral arterial disease

TRIAL	Study population	Study treatment (experimental treatment *vs*. control) and duration	Primary efficacy outcomes	NNT	NNH
**MONOTHERAPY**					
CAPRIE^7^	Patients with prior ischaemic stroke, MI or symptomatic atherosclerotic peripheral arterial disease (*n =* 19 185)	Clopidogrel *vs*. aspirin 1–3 y	Composite of ischaemic stroke, MI or vascular death	196 (104, 5720)	No significant effect
EUCLID^8^	Patients with symptomatic peripheral arterial disease (*n =* 13 885)	Ticagrelor *vs*. clopidogrel Median 30 mo	Composite of cardiovascular death, MI or ischaemic stroke	No significant effect	No significant effect
GLOBAL LEADERS^9^	Patients with stable coronary disease or ACS undergoing PCI (*n =* 15 968)	Aspirin + ticagrelor for 1 mo followed by ticagrelor monotherapy *vs*. aspirin + ticagrelor/clopidogrel for 12 mo followed by aspirin monotherapy	A composite of all‐cause mortality or nonfatal centrally adjudicated new Q‐wave MI at 2 y	No significant effect	No significant effect
TWILIGHT‐ACS^10^	Patients undergoing PCI at high risk of ischaemic or bleeding events (*n =* 7119)	3 mo of aspirin + ticagrelor followed by ticagrelor monotherapy *vs*. 12 mo of aspirin + ticagrelor	Bleeding Academic Research Consortium type 2, 3, or 5 bleeding at 12 mo Secondary endpoint: a composite of death from any cause, nonfatal MI or nonfatal stroke at 12 mo	No significant effect	−32 (−22, −37)
TICO^11^	Patients with ACS treated with drug‐eluting stents (*n =* 3056)	Ticagrelor monotherapy (90 mg twice daily) after 3‐mo DAPT *vs*. ticagrelor‐based 12‐mo DAPT	1‐y net adverse clinical event, defined as a composite of major bleeding and adverse cardiac and cerebrovascular events (death, MI, stent thrombosis, stroke or target‐vessel revascularisation). Secondary endpoint: major adverse cardiac and cerebrovascular events.	50 (29, 222)	No significant effect
**ANTIPLATELET PLUS ANTICOAGULANT THERAPY**					
COMPASS^12^	Patients with history of peripheral artery disease of the lower extremities, of the carotid arteries or coronary artery disease (*n =* 7470)	Aspirin plus rivaroxaban *vs*. aspirin Mean 23 mo	Cardiovascular death, MI or stroke	52 (44, 131)	362 (151, 534)
ATLAS ACS 2‐TIMI 51^13^	Patients with a recent ACS (*n =* 15 526)	Rivaroxaban either 2.5 mg or 5 mg twice daily *vs*. placebo (on top of standard antiplatelet therapy) Up to 31 mo (mean 13 mo)	Composite of death from cardiovascular causes, MI or stroke	63 (19, 453) for 2.5 mg twice daily 53 (43, 468) for 5 mg twice daily	83 (56, 254) for 2.5 mg twice daily 56 (48, 157) for 5 mg twice daily
VOYAGER PAD^14^	Patients with peripheral artery disease who had undergone revascularisation (*n =* 6564)	Aspirin plus rivaroxaban *vs*. aspirin Mean 28 mo	Composite of acute limb ischaemia, major amputation for vascular causes, MI, ischaemic stroke or death from cardiovascular causes	39 (23, 140)	No significant effect
**DAPT**					
CURE^15^	Patients with ACS with non‐STEMI within 24 h from randomisation (*n =* 12 562)	Clopidogrel *vs*. placebo (on a background of aspirin) 3–12 mo	Composite of cardiovascular death, nonfatal MI or stroke at 12 mo	48 (31, 88)	100 (56, 287)
CLARITY^16^	Patients with STEMI (*n =* 3491)	Clopidogrel *vs*. placebo (on a background of aspirin ranging 150–325 mg daily) Patients were to receive study medication daily up to and including the day of coronary angiography. For patients who did not undergo angiography, study drug was to be administered up to and including d 8 or hospital discharge, whichever came first	Death, recurrent MI At 30 d	16 (10, 19)	No significant effect
COMMIT^17^	Patients with MI (93% ST elevation MI, 7% non‐ST elevation MI; *n =* 45 852)	Clopidogrel *vs*. placebo (on a background of aspirin 162 mg) Up to 4 weeks	Death, repeat infarction, stroke At 30 d	111 (71, 330)	No significant effect
DAPT^18^	Patients undergoing PCI with drug‐eluting stent insertion (*n =* 9961)	Following 12 mo of treatment with clopidogrel or prasugrel plus aspirin, patients randomised to continue thienopyridine treatment *vs*. placebo for further 18 mo (on top of aspirin)	Stent thrombosis Major adverse cardiovascular and cerebrovascular events (a composite of death, MI or stroke)	100 (92, 146) 63 (42, 116)	111 (106, 127)
TRITON‐TIMI 38^19^	Patients with ACS scheduled for PCI (*n =* 13 608)	Prasugrel *vs*. clopidogrel (on a background of aspirin 75–162 mg) 6–15 mo	Cardiovascular death, MI or stroke	45 (32, 87)	166 (89, 500)
PLATO^20^	Patients with ACS within 24 h from randomisation (*n =* 18 624)	Ticagrelor *vs*. clopidogrel (on a background of aspirin 75–100 mg) 12 mo	A composite of death from vascular causes, MI or stroke at 12 mo	53 (12, 115)	No significant effect
PEGASUS‐TIMI 54^21^	Patients who had had a MI 1 to 3 y previously (*n =* 21 162)	Ticagrelor 90 mg twice daily *vs*. ticagrelor 60 mg twice daily *vs*. placebo Median 33 mo	Composite of cardiovascular death, MI or stroke	85 (49, 306) for 90 mg 82 (47, 245) for 60 mg	65 (48, 135) for 90 mg 80 (59, 191) for 60 mg
THEMIS‐PCI^22^	Patients 50 y or older, with type 2 diabetes receiving antihyperglycaemic drugs for at least 6 mo, with stable coronary artery disease, and previous PCI (*n =* 11 154)	Ticagrelor *vs*. placebo (on a background of aspirin) Median 3.3 y	Composite of cardiovascular death, MI or stroke (median follow up 3.3 y)	77 (45, 389)	111 (51, 187)
HOST‐EXAM^23^	Patients on DAPT without clinical events for 6–18 mo after PCI with drug‐eluting stents or aspirin 100 mg once daily for 24 mo (*n =* 5438)	Monotherapy with clopidogrel 75 mg daily *vs*. aspirin 100 mg daily 24 mo	Composite of all‐cause death, nonfatal MI, stroke, readmission due to ACS, and Bleeding Academic Research Consortium bleeding type 3 or greater at 24 mo	50 (32, 132)	‐ (harm from bleeding included in primary composite endpoint)

NNT: number needed to treat for primary efficacy outcome (with 95% confidence intervals). Negative value indicates control treatment more efficacious on primary outcome than experimental treatment.

NNH: number needed to harm for primary safety outcome (with 95% confidence intervals). Negative value indicates control treatment gives more harm than experimental treatment.

Values for NNT and NNH are only given for clinical outcomes and if the difference in efficacy or harm attained statistical significance in the study.

ACS, acute coronary syndrome; MI, myocardial infarction; PCI, percutaneous coronary intervention.

## 3 SEARCH STRATEGY AND SELECTION CRITERIA

We searched PubMed for relevant articles published in the English language between 1 January 2000 and 30 August 2021 using the terms aspirin, clopidogrel, prasugrel, ticagrelor, clinical, antiplatelet, guidelines, randomised clinical trials, systematic reviews, meta‐analyses. We focused on literature published in the past 5 years but make reference to earlier studies where relevant.

## 4 PRIMARY PREVENTION

Despite the now well‐established role of aspirin in secondary cardiovascular prophylaxis, the benefit/risk ratio in primary prevention is far less clear. In low‐ and middle‐income countries, aspirin‐containing polypill strategies have proved effective in preventing major cardiovasacular events, for example in the PolyIran study.^24^ However, a large meta‐analysis conducted by the Antithrombotic Trialists' collaboration from 2009 questioned the net benefit of aspirin in primary prevention as a result of an observed increased risk of major extracranial and gastrointestinal bleeding complications in spite of only a small protective effect against vascular events.^25^ These findings have been confirmed by the most recent trials conducted in primary prevention: for example, ASPREE, which focused on elderly subjects, ASCEND, which studied patients with diabetes, and ARRIVE, which examined patients with a moderate estimated risk of a first cardiovascular event,^26^, ^27^, ^28^ showing that the net benefit of aspirin in this setting is marginal at best whilst posing a major bleeding hazard in subjects with cardiovascular risk factors who are otherwise healthy. At present, therefore, a cautious approach is advised as regards the use of aspirin in primary prevention, weighing the benefit to risk ratio in order to personalise treatment.

## 5 SECONDARY PREVENTION: CORONARY ARTERY DISEASE

According to the most recent European guidelines,^29^, ^30^ patients with coronary artery disease are categorised into acute coronary syndrome (ACS) and chronic coronary syndrome (CCS) groups, depending on the clinical scenario. Aspirin remains the first line option as monotherapy for long‐term (>12 months) treatment in all categories of patients in sinus rhythm, whilst anticoagulants should be considered in the presence of atrial fibrillation given their demonstrated superiority over aspirin for reduction of cardioembolic stroke that are the main cause of mortality and morbidity in patients with atrial fibrillation. Although no data currently exist to support DOAC monotherapy in ACS or CCS, in patients with coronary disease and concomitant atrial fibrillation at high bleeding risk (as assessed by HAS‐BLED score) where monotherapy is considered desirable, the choice between antiplatelet and DOAC therapy will depend on the relative risk of coronary plaque rupture or stent thrombosis *vs*. that of stroke (as determined by CHA₂DS₂‐VASc score). In patients with combined coronary disease and atrial fibrillation at low bleeding risk, combination antiplatelet and anticoagulant therapy may be considered, in which situation evidence supports the use of clopidogrel and a DOAC, rather than regimens that include a vitamin K antagonist, aspirin, or both, due to less bleeding and fewer hospitalisations without significant differences in the incidence of ischaemic events; this includes patients post‐ACS or percutaneous coronary intervention (PCI).^31^

The only available direct comparison of clopidogrel *vs*. aspirin in the context of CCS is provided by the CAPRIE study (Table 1),^7^ that demonstrated 8.7% relative risk reduction with clopidogrel in the composite outcome of ischaemic stroke, myocardial infarction (MI) or vascular death in the overall population. However, within the subgroup of patients with prior MI, the 2 antiplatelet agents performed similarly, with in fact an apparent but nonsignificant advantage of aspirin over clopidogrel (3.7% relative risk reduction in favour of aspirin). Aspirin has traditionally been used over clopidogrel as monotherapy for historical rather than efficacy reasons, as well as its slightly lower cost. However, the recently published HOST‐EXAM trial (Table 1) demonstrated, in patients who had received DAPT for 6–18 months after PCI with drug‐eluting stents, that subsequent monotherapy with clopidogrel 75 mg daily reduced the risk of the composite of all‐cause death, nonfatal MI, stroke, readmission due to ACS, and Bleeding Academic Research Consortium bleeding type 3 or greater compared to aspirin 100 mg daily, suggesting that clopidogrel may be superior to aspirin monotherapy in this situation.^23^

### 5.1 ACS

Clopidogrel is approved as add‐on therapy to aspirin in the context of a DAPT regimen following ACS, as supported by the CURE trial in non‐ST elevation ACS,^15^ the CLARITY trial in ST‐elevation MI (STEMI),^16^ and the COMMIT trial in STEMI^17^ (Table 1). A consistent finding across these trials was the beneficial effect of clopidogrel as add‐on therapy to aspirin in reducing future MI, whilst the preventative action of the drug combination on stroke was marginal.

Prasugrel and ticagrelor are not licensed as monotherapy for routine long‐term antithrombotic prophylaxis, because trials that have tested their benefit in this clinical setting are lacking, and their more intense antiplatelet action is likely to increase haemorrhagic complications. More intensive platelet inhibition, as achieved with prasugrel or ticagrelor in combination with aspirin, is justified when the risk of cardiovascular events is deemed particularly high and/or prior antithrombotic therapies have failed, as in patients who experience events or stent thrombosis while on clopidogrel, or in the periprocedural period of PCI following either an acute event or elective stenting with unfavourable risk factors such as diabetes or left main stenting. Of note, prasugrel is authorised only following PCI, since the registration trial TRITON‐TIMI 38 (Table 1) specifically tested the drug in patients with ACS scheduled for PCI.^19^ By contrast, ticagrelor is indicated for ACS whether treated medically or by PCI, since efficacy was seen in both settings.^20^

As regards standard DAPT, current guidelines^29^, ^30^ endorse treatment for 6–12 months, followed by aspirin monotherapy. A shorter duration of DAPT should be considered in patients at high bleeding risk, whilst DAPT may be extended beyond 1 year in patients at high ischaemic risk (e.g. stent thrombosis, recurrent ACS on DAPT, post‐MI/diffuse disease) as long as the bleeding potential is low (e.g. no prior bleeding on DAPT, coagulopathy or oral anticoagulant use). Both prasugrel and ticagrelor are superior to clopidogrel for prevention of thrombotic events, although their higher antiplatelet efficacy is counterbalanced by enhanced bleeding risk. In particular, subgroup analyses within TRITON‐TIMI 38 identified several categories of patients for whom the benefit‐to‐risk ratio of prasugrel appears unfavourable, so that warnings have been issued for its use in patients with body weight ≤60 kg, those with a history of stroke or transient ischaemic attack (TIA), and those over the age of 75 years. As to choice between these 2 agents where there are no clear indications for 1 over the other, the ISAR‐REACT 5 trial demonstrated that, among patients with ACS with or without ST‐segment elevation, the incidence of death, myocardial infarction, or stroke was significantly lower with prasugrel than with ticagrelor, with no difference in major bleeding.^32^ As a result, the most recent European Society of Cardiology guidelines on management of non‐ST‐segment elevation ACS gave a strong level of recommendation (IIa) in favour of prasugrel over ticagrelor in these patients.^30^

In the GLOBAL LEADERS trial (Table 1),^9^ ticagrelor given in combination with aspirin for 1 month followed by 23 months of ticagrelor monotherapy failed its primary superiority outcome on safety compared to 12 months of standard DAPT followed by 12 months of aspirin alone. By contrast, the TWILIGHT‐ACS study^10^ (Table 1) reported an advantage of ticagrelor monotherapy initiated after 3 months of combined therapy with aspirin *vs*. standard DAPT (i.e. 12 months ticagrelor and aspirin co‐administration), on the basis of a lower incidence of clinically relevant bleeding events in patients at high risk for bleeding or ischaemic events undergoing drug‐eluting stent implantation, without compromising prevention of death, myocardial infarction, or stroke. Moreover, the TICO trial (Table 1) demonstrated that, in patients with ACS treated with drug‐eluting stents, ticagrelor monotherapy after 3 months of DAPT, compared with ticagrelor‐based 12‐month DAPT, resulted in a reduction in the composite outcome of major bleeding and cardiovascular events at 1 year.^11^ Although these studies utilised an alternative P2Y_12_ inhibitor, their findings reinforce the previously generated evidence with clopidogrel in combination with aspirin lasting 1–3 months followed by clopidogrel monotherapy.^33^, ^34^ Therefore, 3 months of DAPT followed by P2Y_12_ inhibitor monotherapy may have advantages over the standard 6–12 months of DAPT followed by aspirin monotherapy, in patients with ACS (either those treated by PCI or by medical therapy) as well as in patients undergoing elective PCI. Furthermore, data from the Patterns of Non‐Adherence to Antiplatelet Regimens in Stented Patients (PARIS) registry suggests that physician‐guided discontinuation of DAPT is safe and not associated with increased risk of major adverse cardiac events,^35^ thus supporting the place of tailoring of DAPT according to individual patient characteristics including bleeding risk.

### 5.2 CCS

As discussed above, patients with CCS are generally treated with antiplatelet monotherapy, usually aspirin; although in patients with previous ACS emerging evidence suggests that, following an initial period of DAPT, clopidogrel monotherapy may be superior. For patients with CCS and peripheral arterial disease (PAD), dual pathway inhibition with the combination of aspirin and low‐dose rivaroxaban was recently approved for long‐term prophylaxis, owing to the results of the COMPASS trial showing a reduction of the primary outcome (a composite of stroke, MI and cardiovascular death) with the combined therapy compared to aspirin monotherapy.^12^ Analysis of the individual components of the composite endpoint revealed a major impact of this drug combination on prevention of ischaemic stroke (hazard ratio 0.51 [0.38–0.68]; *P* < .001), while the effect on MI prevention was nonsignificant. Notably, there was a small increase in bleeding with the dual pathway inhibition strategy compared to aspirin alone (hazard ratio 1.70; 95% confidence interval 1.40–2.05; *P* < .001), without a corresponding increase in intracranial or fatal bleeding.

Although the standard dose of ticagrelor is 90 mg twice daily when given with aspirin as part of DAPT in the context of ACS, the lower dose of 60 mg twice daily has been investigated in the PEGASUS‐TIMI 54 trial (Table 1), compared to the standard 90 mg twice daily dose or placebo, on a background of aspirin in patients with a history of MI 1–3 years previously.^21^ Both ticagrelor doses gave rise to a reduction in risk of cardiovascular death, MI or stroke, as well as an increase in thrombolysis in MI (TIMI) major bleeding; and although the increased bleeding risk was numerically lower in the 60‐mg group, this was not significant. However, the lower dose appeared to be associated with reduced side effects, in particular dyspnoea. Ticagrelor 60 mg twice daily on top of aspirin may therefore be a valuable treatment option for patients with a prior history of MI who are at high risk of an atherothrombotic event, following the initial period of standard DAPT after their acute presentation.

## 6 CEREBROVASCULAR DISEASE

Unlike cardiac disease, the range of different subtypes of cerebrovascular disease^36^ provides an additional layer of complexity to its management. Our evolving understanding of genetic differences in certain stroke subtypes^37^ provides the possibility of more focused interventions in the future, although subtype‐specific clinical trials using antiplatelet medication have not been undertaken.

The use of antiplatelet monotherapy for secondary stroke prevention is well established, from the CAST and IST studies.^38^, ^39^ The possible place of DAPT in the prophylaxis of cerebrovascular events has been much investigated in attempting to improve the effectiveness of aspirin or clopidogrel monotherapy. The combination of aspirin and clopidogrel has been tested in the MATCH,^40^ SPS3^41^ and CHARISMA^42^ trials (Table 2); in none of these did DAPT demonstrate superiority over antiplatelet monotherapy in preventing recurrent ischaemic strokes, despite increased bleeding complications, and, in MATCH, clopidogrel monotherapy yielded the best outcomes, although these trials recruited patients not necessarily with recent stroke.

**TABLE 2:** Major randomised controlled clinical trials testing antiplatelet strategies in the secondary prophylaxis of cerebrovascular disease

TRIAL	Study population	Study treatment (experimental treatment *vs*. control) and duration	Primary efficacy outcomes	NNT	NNH
**MONOTHERAPY**					
CAPRIE^7^	Patients with prior ischaemic stroke, myocardial infarction, or symptomatic atherosclerotic peripheral arterial disease (*n =* 19 185)	Clopidogrel *vs*. aspirin 1–3 y	Composite of ischaemic stroke, myocardial infarction, or vascular death	196 (104, 5720)	No significant effect
PROFESS^43^	Patients with stroke or TIA (*n =* 20 333)	Clopidogrel *vs*. Aspirin + dipyridamole Mean 2.5 y	Recurrent stroke at 2.5 y	No significant effect	−200 (−76, −500)
SOCRATES^44^	Patients with nonsevere ischaemic stroke or high‐risk TIA who had not received thrombolysis and were not considered to have had a cardioembolic stroke (*n =* 13 199)	Ticagrelor *vs*. aspirin 90 d	Time to the occurrence of stroke, myocardial infarction or death within 90 d	No significant effect	No significant effect
**ANTIPLATELET PLUS ANTICOAGULANT THERAPY**					
COMPASS^12^	Patients with history of peripheral artery disease of the lower extremities, of the carotid arteries or coronary artery disease (*n =* 7470)	Aspirin plus rivaroxaban *vs*. aspirin Mean 23 mo	Cardiovascular death, myocardial infarction or stroke; the primary peripheral artery disease outcome was major adverse limb events including major amputation	52 (44, 131)	362 (151, 534)
VOYAGER PAD^14^	Patients with peripheral artery disease who had undergone revascularisation (*n =* 6564)	Aspirin plus rivaroxaban *vs*. aspirin Mean 28 mo	Composite of acute limb ischaemia, major amputation for vascular causes, myocardial infarction, ischaemic stroke or death from cardiovascular causes	39 (23, 140)	No significant effect
**DUAL ANTIPLATELET THERAPY**					
MATCH^40^	Patients with multiple risk factors and symptomatic disease (*n =* 7599)	Aspirin *vs*. placebo (on a background of clopidogrel 75 mg) 18 mo	Composite of ischaemic stroke, myocardial infarction, vascular death, rehospitalisation at 18 mo	No significant effect	77 (53, 278)
SPS3^41^	Patients with recent symptomatic lacunar infarcts (*n =* 3020)	Clopidgrel *vs*. placebo (on a background of aspirin 325 mg) Mean 3.4 y	Any recurrent stroke, including ischaemic stroke and intracranial haemorrhage at 3.4 mo	No significant effect	31 (16, 65)
CHARISMA^42^	Patients with multiple risk factors or evident cardiovascular disease (*n =* 15 603)	Clopidogrel *vs*. placebo (on a background of aspirin 75–162 mg) Median 28 mo	A composite of cardiovascular death, myocardial infarction, or stroke at 28 mo	No significant effect	No significant effect
CHANCE^45^	Patients with minor ischaemic stroke or high‐risk TIA within 24 h from randomisation (*n =* 5170)	Clopidogrel *vs*. placebo (on a background of aspirin 75–300 mg) 90 d	Stroke (ischaemic or haemorrhagic) at 90 d	28 (15, 45)	No significant effect
POINT^46^	Patients with minor ischaemic stroke or high‐risk TIA within 24 h from randomisation (*n =* 4881)	Clopidogrel *vs*. placebo (on a background of aspirin 50–325 mg) 90 d	Composite of ischaemic stroke, myocardial infarction, or death from an ischaemic vascular event, at 90 d	64 (37, 306)	186 (63, 500)
ESPS2^47^	Patients with TIA or stroke within the preceding 3 mo (*n =* 6602)	Aspirin (25 mg twice daily) modified‐release plus dipyridamole 200 mg twice daily *vs*. aspirin 2 y	Fatal and nonfatal stroke; death from any cause; stroke and/or death at 24 mo	33 (27, 51) for stroke	Data not available
ESPRIT^48^	Patients with TIA or minor stroke within the last 6 mo (*n =* 2739)	Aspirin plus dipyridamole *vs*. aspirin (30–325 mg) Median 3.5 y	Composite of death from all vascular causes, nonfatal stroke, nonfatal myocardial infarction, or major bleeding complications at 5 y	33 (19, 319)	77 (40, 500)
CARESS^49^	Patients with carotid territory TIA (including amaurosis fugax) or stroke within the last 3 mo, with microembolic signals on transcranial doppler ultrasound (*n =* 107)	Clopidogrel plus aspirin *vs*. aspirin 7 d	Proportion of patients with microembolic signals present at d 7	Clinical efficacy endpoints not measured (39.8% reduction in microembolic signals)	‐
FASTER^50^	Patients with TIA or minor stroke (*n =* 392)	Clopidogrel plus aspirin *vs*. aspirin 90 d	Total stroke (ischaemic and haemorrhagic) within 90 d	No significant effect	No significant effect
ECLIPse^51^	Patients with acute lacunar infarction (*n =* 203)	Cilostazol plus aspirin *vs*. aspirin 90 d	Changes of middle cerebral artery and basilar artery pulsatility index (measured by transcranial doppler ultrasound) at 14 and 90 d from baseline	Clinical efficacy endpoints not measured (decrease in pulsatility index)	‐
COMPRESS^52^	Patients with acute ischaemic stroke due to large‐vessel atherosclerotic disease within 48 h (*n =* 358)	Aspirin plus clopidogrel *vs*. aspirin	Change in ischaemic lesion burden on magnetic resonance imaging	Clinical efficacy endpoints not measured (no difference in new ischaemic lesions)	‐
PRINCE^53^	Patients with acute minor stroke or TIA (*n =* 675)	Ticagrelor *vs*. clopidogrel (on a background of aspirin) 90 d	Change in proportion of patientswith high platelet reactivity at 90 d	No difference in stroke recurrence (but this was a secondary endpoint). 60% reduction seen in proportion of patients with high platelet reactivity.	No difference
THALES^54^	Patients who had had a mild‐to‐moderate acute noncardioembolic ischaemic stroke, with a National Institutes of Health stroke scale score of 5 or less, or TIA and who were not undergoing thrombolysis or thrombectomy (*n =* 11 016)	Ticagrelor plus aspirin *vs*. aspirin 30 d	Composite of stroke or death within 30 d	91 (52, 379)	250 (108, 500)

NNT: number needed to treat for primary efficacy outcome (with 95% confidence intervals). Negative value indicates control treatment more efficacious on primary outcome than experimental treatment.

NNH: number needed to harm for primary safety outcome (with 95% confidence intervals). Negative value indicates control treatment gives more harm than experimental treatment.

Values for NNT and NNH are only given for clinical outcomes and if the difference in efficacy or harm attained statistical significance in the study.

TIA, transient ischaemic attack.

By contrast, the CHANCE^45^ and POINT^46^ studies (Table 2) have provided evidence supporting a short course of DAPT in patients with minor ischaemic stroke and TIA. Both trials demonstrated an advantage of the combined therapy on clinical outcomes (a composite of ischaemic stroke, MI or death measured at 90 d). However, POINT but not CHANCE reported a higher rate of major bleeding complications; and, in a secondary analysis, the benefit of DAPT was apparent predominantly during the first 21 days of therapy.^55^ The recently published THALES trial (Table 2) showed that, among patients with a mild‐to‐moderate acute noncardioembolic ischaemic stroke or TIA who were not undergoing thrombolysis, the risk of the composite of stroke or death within 30 days was lower with combination ticagrelor and aspirin than with aspirin alone, but the incidence of disability did not differ significantly between the 2 groups; severe bleeding was more frequent with ticagrelor.^54^

Therefore, unlike the prophylaxis of cardiac events, secondary prevention of cerebral events by DAPT has shown advantage over monotherapy only in short‐term therapy and for patients with minor stroke or TIA. This is probably due to the increased probability of reoccurrence of a major stroke, often disabling, within 2 weeks from the first event^56^; the bleeding risk associated with antithrombotic therapies is generally early from therapy initiation, although it may decline after the first month.^57^

For long‐term prophylaxis, the combination of aspirin and dipyridamole can be considered as long as it is tolerated. Two major trials tested this combination, namely the ESPS2^47^ and ESPRIT^48^ studies (Table 2), demonstrating advantage in terms of a composite endpoint of death from all vascular causes, stroke and MI for aspirin plus dipyridamole over aspirin alone, without significant impact on haemorrhagic risk. However, a high therapy discontinuation rate (about 6%)^58^ has been reported for this combination, which appears to be related to the occurrence of headache.

Where monotherapy is considered for long term prophylaxis, clopidogrel is preferred over aspirin on the basis of both the CAPRIE results,^7^ comparing clopidogrel to aspirin, and the PROFESS trial,^43^ testing clopidogrel against aspirin plus dipyridamole, which respectively reported greater protection with clopidogrel than with aspirin and similar protection to aspirin plus dipyridamole, against a composite of ischaemic stroke, myocardial infarction, or vascular death (Table 2). These results were recently confirmed by a meta‐analysis of 5 trials including CAPRIE.^59^ Triple antiplatelet therapy of aspirin, clopidogrel and dipyridamole is not recommended in secondary prevention of stroke as it does not enhance protection but significantly increases the risk of major bleeding.^60^

Hence, current guidelines suggest either clopidogrel or aspirin plus dipyridamole as equivalent alternatives for long‐term secondary prophylaxis of stroke.

## 7 PERIPHERAL ARTERIAL DISEASE

There is no clear consensus between different international guidelines on antithrombotic therapy in stable PAD. Data in this situation largely derive from subanalyses of randomised trials of patients with various manifestations of atherosclerosis, including coronary disease, cerebrovascular disease and PAD. Currently there is no convincing evidence for efficacy of any antithrombotic strategy in patients with asymptomatic PAD whereas, by contrast, the evidence of benefit is much clearer in those with symptomatic PAD. Single antiplatelet therapy with either aspirin or clopidogrel is well established to reduce cardiovascular risk, and more recently data from the COMPASS trial support combined therapy with aspirin and rivaroxaban in this situation.^12^ Patients who undergo either surgical or endovascular revascularisation for PAD should be prescribed lifelong antithrombotic therapy; and dual antithrombotic therapy with aspirin plus rivaroxaban is recommended, on the basis of the recently published VOYAGER PAD trial (Table 1),^14^ which demonstrated that addition of rivaroxaban 2.5 mg twice a day to aspirin in such patients reduced the relative incidence of the composite outcome of acute limb ischaemia, amputation for vascular causes, MI, ischaemic stroke or cardiovascular death by 15%, with no significant increase in TIMI major bleeding, compared to aspirin alone.

## 8 RECENT UPDATES FOR SELECTED PATIENT SUBGROUPS

### 8.1 Kidney disease

Kidney disease is considered a coronary heart disease risk equivalent, and as such it carries a particularly high cardiovascular risk according to guidelines.^29^, ^30^ The use of antiplatelet drugs in patients with chronic kidney disease accordingly follows the recommendations that apply to patients in the very high cardiovascular risk category, even though evidence in support of therapy decision making is limited by a paucity of data, especially for patients with end‐stage renal disease and those on dialysis who were often excluded from trials. Although some evidence had suggested an association between aspirin use and increased risk of MI in patients on haemodialysis,^61^ and another observational study had reported increased mortality associated with antithrombotic treatment in patients with kidney disease,^62^ despite lack of randomised controlled trial data, the weight of evidence suggests that antiplatelet treatment (used in accordance with current guidelines for patients at very high cardiovascular risk) is beneficial in patients with advanced kidney disease, the overall benefits outweighing the risks; but that a carefully tailored approach should be taken where the bleeding risk is judged to be especially high in an individual.

As discussed above, dual pathway inhibition with aspirin and rivaroxaban is now licensed for treatment of patients with CCS and PAD. However, since rivaroxaban (and indeed other DOACs) are predominantly excreted renally, the rivaroxaban plus aspirin combination should be used with caution in patients with kidney disease.

### 8.2 Diabetes

Given the known increase in prothrombotic status conferred by diabetes, antiplatelet prophylaxis was widely used in patients with diabetes (both types 1 and 2) for primary prevention until evidence from the Antithrombotic Trialists' Collaboration Collaborative meta‐analysis indicated lack of benefit of aspirin in the absence of established cardiovascular disease. Antiplatelet drugs are now prescribed, as in nondiabetics, in patients with prior cardiovascular events and/or established disease.^29^, ^30^ Of note, the benefit of DAPT post‐PCI appeared to be more pronounced in diabetics than nondiabetics in TRITON‐TIMI 38 (17 *vs*. 12.2% relative reduction of ischaemic events in the respective groups), based on which prasugrel is now indicated by guidelines for DAPT in diabetic patients post‐PCI.^30^

The THEMIS trial^63^ explored the potential advantage of adding ticagrelor to aspirin in the long‐term treatment of diabetics with known stable coronary disease but without prior events. Although there was superiority in the reduction of a composite of MI, stroke and cardiovascular death, the primary safety outcome of major bleedings was unfavourable for the combined therapy compared to aspirin monotherapy, making the effect on the exploratory outcome of irreversible harm neutral (death from any cause, MI, stroke, fatal bleeding, or intracranial haemorrhage).

In short, although it is clear that patients with diabetes (either type 1 or type 2) carry increased cardiovascular risk, the weight of evidence suggests that antiplatelet therapy should be utilised in such patients in exactly the same way as in nondiabetics. Although recent joint European Society of Cardiology/European Association for the Study of Diabetes guidelines have proposed that cardiovascular risk level in patients with diabetes should be classified as moderate, high and very high (so that no low risk category exists in such patients), the recommendation remains unchanged that antiplatelet therapy should be prescribed according to primary or secondary prevention, just as for nondiabetic patients.^64^

### 8.3 Elderly

Age in itself elevates cardiovascular risk regardless of additional risk factors. Additionally, there is a continuum in the age‐dependent increase in bleeding hazard from antiplatelet agents, such that age has been included among the main clinical variables of scores recommended by international guidelines to calculate bleeding risk at the individual level,^65^ such as the DAPT (which assesses ischaemic and bleeding risks at 12–30 mo following PCI) and PRECISE‐DAPT (a simple 5‐item risk score that predicts out‐of‐hospital bleeding during DAPT) scores.

TRITON‐TIMI 38 reported unfavourable outcomes with prasugrel (compared to clopidogrel) combined with aspirin post‐PCI in elderly people, making age 75 years or over a contraindication to prasugrel treatment due to unacceptable bleeding hazard. A reduced dose of prasugrel was tested specifically in the elderly and compared to clopidogrel in the TRILOGY ACS substudy^66^ and ELDERLY ACS 2 trial,^67^ the former demonstrating similar performance in terms of efficacy and safety outcomes between the 2 regimens, and the latter interrupted early for futility. A reduced length of treatment less than the conventional 12 months has also been the subject of investigation in the SENIOR clinical trial,^68^ which showed that drug‐eluting stent implantation and 1 or 6 months of DAPT in patients with stable or unstable clinical presentation, respectively, offer similar outcomes to bare metal stent implantation and 12 months of standard DAPT, suggesting that a short course of DAPT and drug‐eluting stent insertion may minimise bleeding risk in elderly patients undergoing PCI. A step‐down approach has been trialled in the elderly in the ANTARCTIC^69^ and TROPICAL‐ACS^70^ studies, using platelet function testing to de‐escalate patients from prasugrel to clopidogrel on a background of aspirin over a 12‐month period. None of these studies reported convincing data in support of such a strategy to maximise benefit while reducing bleeding risk. A sub‐study of PLATO,^71^ comparing ticagrelor to clopidogrel in DAPT, showed that the benefit of the former was independent of age. However, the recently published POPular AGE trial^72^ questioned these data by reporting that clopidogrel exerts a similar antithrombotic activity to more potent antiplatelet drugs in the elderly with a reduced incidence of bleeding.

On the basis of current evidence, therefore, elderly patients with established atherosclerotic disease should receive antiplatelet monotherapy or DAPT in the same circumstances as younger patients, with the proviso that clopidogrel may be preferred to prasugrel or ticagrelor as part of DAPT.

## 9 OTHER APPROACHES TO ANTIPLATELET THERAPY STRATIFICATION

As highlighted above, currently treatment decisions around length and intensity of antiplatelet therapy are guided by clinical judgements—including risk scores—around thrombotic *vs*. haemorrhagic risk in individual patients, including those within the special groups outlined above. Other strategies to better personalise antiplatelet therapy are being researched.

### 9.1 Genotyping

Cytochrome P450 (CYP) allelic variant genotyping has long been considered potentially important in guiding selection of P2Y_12_ inhibitors, as a result of of the requirement for the thienopyridines to undergo CYP‐mediation conversion to their active metabolites (Figure 1) and of the demonstrated effects of CYP variants on clopidogrel pharmacokinetics and pharmacodynamics. It is well established that carriers of CYP2C19 loss‐of‐function (LOF) variants exhibit reduced exposure to the active metabolite of clopidogrel compared to noncarriers^73^, ^74^ and hence impaired suppression of platelet activity by clopidogrel.^75^ In light of this, prospective randomised studies have been undertaken to investigate the clinical utility of genotype‐based antiplatelet therapy selection. The POPular Genetics study^76^ was conducted in STEMI patients undergoing PCI who were assigned to receive either a P2Y_12_ inhibitor on the basis of early CYP2C19 LOF genetic testing (genotype‐guided group) or standard treatment with either ticagrelor or prasugrel (standard‐treatment group) for 12 months. The results showed no difference in the composite outcome of MI, stroke and cardiovascular death, with superiority of safety (decrease in the primary bleeding endpoint), in the genotype‐guided group. In the TAILOR‐PCI study,^77^ which studied patients with either stable or unstable coronary disease undergoing PCI randomised to a standard approach (with clopidogrel and no genotyping) or genotype‐based therapy with clopidogrel or ticagrelor in LOF allele noncarriers and carriers, respectively, the composite end point of cardiovascular death, MI, stroke, stent thrombosis or severe recurrent ischaemia at 12 months was 4.0% in the genotype‐based therapy and 5.9% in the standard approach groups respectively, not quite reaching statistical significance (hazard ratio 0.66, [95% confidence interval, 0.43–1.02]; *P* = .06).

The PLATO substudy^78^ suggested clinical relevance of the CYP2C19 polymorphisms on response to therapy, as did the PHARMCLO trial,^79^ although this latter used a broadened genotyping strategy to include genes other than CYP2C19. Indeed, polymorphisms affecting proteins involved in absorption of drugs such as P‐glycoprotein could play a role as well. As regards cerebrovascular disease, a meta‐analysis reported a relationship between CYP2C19 poor metaboliser alleles and efficacy of clopidogrel in secondary prevention of stroke/TIA.^80^ However, randomised interventional trials are not available in this context. Moreover, little information is available about the potential clinical relevance of gene polymorphisms on prasugrel and ticagrelor therapy.

Therefore, although a genotyping approach holds promise, to date its value in guiding choice of antiplatelet therapy remains unclear in terms of clinical outcomes.

### 9.2 Platelet function testing

A number of randomised controlled trials in coronary heart disease including the GRAVITAS,^81^ TRIGGER‐PCI^82^ and ANTARCTIC^69^ studies have not shown a clear clinical advantage of selecting therapy, with particular reference to P2Y12 antagonists as part of DAPT, based on functional platelet assays. To evaluate the impact of platelet testing in real world practice, the TRANSLATE‐POPS study^83^ investigated the usefulness of implementing platelet function studies for ACS patients undergoing PCI, but showed no effect on either 30‐day cardiovascular outcomes or bleeding. Other studies suggest that platelet function testing can result in improved outcomes, such as reduced stent thrombosis following PCI.^84^

Similarly, in cerebrovascular disease, the PRINCE trial (Table 2) showed that patients with minor stroke or TIA treated with ticagrelor plus aspirin exhibit reduced platelet reactivity compared to those receiving clopidogrel plus aspirin, especially so in carriers of the CYP2C19 LOF allele.^53^ Whether this translates into improved clinical outcomes remains unknown.

At present, therefore, platelet function testing remains of uncertain usefulness for treatment decision making, although research continues to explore whether better definition of patient groups in whom such testing might be beneficial might improve its applicability.

## 10 CONCLUSIONS

Antiplatelet therapy is an important component of cardiovascular and cerebrovascular prophylaxis, in patients with documented atherosclerotic disease; and modern antiplatelet agents, alone or in combination, can powerfully inhibit thrombotic complications. However, intensive platelet inhibition carries the price of increased haemorrhagic risk, and the risk of serious, even life‐threatening, bleeding in predisposed patients. Therefore, in all patients, a careful assessment of thrombotic *vs*. bleeding risk must be made, and antiplatelet therapy tailored accordingly. Certain patient populations require particular considerations as regards antiplatelet therapy.

A frequent clinical concern is what to do as regards antiplatelet therapy (both its use and its intensity) in patients with a history of prior bleeding. The clinician's judgement in such situations should take into account the site and severity of that bleeding, as well as how long ago it happened, what the circumstances/precipitants were, and whether the underlying cause was adequately treated. As we have discussed throughout the article, the most important consideration for the clinician should be the risk of future, as opposed to simply a history of previous, bleeding.

Genotyping and platelet function testing allow ever more accurate prediction of the effects of antiplatelet therapies on platelet function in vitro. At present, use of these has not been clearly demonstrated to translate into clinical utility, although further research is needed to clarify whether they may be of use in certain better defined patient populations.

### 10.1 Nomenclature of targets and ligands

Key protein targets and ligands in this article are hyperlinked to corresponding entries in http://www.guidetopharmacology.org, and are permanently archived in the Concise Guide to PHARMACOLOGY 2019/20.^85^, ^86^

## COMPETING INTERESTS

The authors declared no conflicts of interest.
